# Human and natural factors affect habitat quality in ecologically fragile areas: evidence from Songnen Plain, China

**DOI:** 10.3389/fpls.2024.1444163

**Published:** 2024-11-19

**Authors:** Xiuli Sun, Yuehui Li, Yuanman Hu, Yue Li

**Affiliations:** ^1^ School of Environment, Liaoning University, Shenyang, China; ^2^ CAS Key Laboratory of Forest Ecology and Silviculture, Institute of Applied Ecology, Chinese Academy of Sciences, Shenyang, China

**Keywords:** ecologically fragile areas, Songnen Plain, habitat quality, InVEST model, structural equation model, influencing factors

## Abstract

Habitat quality (HQ) has been progressively degrading worldwide in recent decades due to rapid climate change and intensive human activities. These changes not only threaten biodiversity and ecosystem functions, but also impact socio-economic development. Therefore, a few studies have focused on the dynamics of HQ and its natural and anthropogenic drivers. However, many contributions have failed to reveal how these factors interact to impact HQ, especially in ecologically fragile areas. We estimated HQ in the Songnen Plain of Northeast China, an ecologically fragile area, from 2000 to 2020 using the InVEST model and explored the response of HQ to the interactions of natural factors (topography, climate, NDVI) and anthropogenic factors (nighttime light index, population density) influencing HQ using Structural Equation Modelling (SEM). The results showed that 1) HQ decreased constantly from 2000 to 2018, and then increased slightly from 2018 to 2020. 2) In terms of spatial distribution, HQ appeared to be highly heterogeneous with a pattern of ‘high HQ in the east – low HQ in the center – high HQ in the west’ at each time point. The high-HQ areas were significantly clustered in the eastern parts with dense forests, while the low-HQ areas in the central parts were dominated by a large number of man-made patches of agriculture and towns or cities. 3) The spatial patterns of HQ are mainly affected by the interactions of factors including the natural environment and human disturbance. Natural factors had a greater impact on HQ than human disturbance, and human disturbance factors had significant negative impact among all these factors at 4 time points. Furthermore, the intensity of the impact of various influencing factors on habitat quality, as well as the positive or negative effects of these drivers on habitat quality, changed over time. The most important influencing factor was temperature in 2000 and topography in 2010, 2018, and 2020. This study can provide important suggestions for future ecological protection and restoration in similar ecologically fragile areas.

## 1 Introduction

Habitat quality (HQ) refers to the ability of ecosystems to provide suitable living conditions for species to survive and reproduce (Bai et al., 2019; Wu et al., 2022; Zhao et al., 2022c),which reflects the level of biodiversity, services and health of ecosystems (Zhu et al., 2020; Li et al., 2022). However, multiple disturbances such as climate change, high population growth and rapid economic development have increasingly degraded the natural environment worldwide, leading to a decline in biodiversity and subsequent degradation of habitat quality (Yohannes et al., 2021; Ti et al., 2023). Therefore, it is essential and urgent to make appropriate decisions to improve habitat quality and ultimately promote sustainable development. To achieve this goal, it is crucial to assess the spatial and temporal changes in HQ, identify the driving factors behind the changes, and detect the interactions among these factors in these degraded areas at the regional scale (Wang et al., 2011; Miguel et al., 2018).

Existing studies assessing HQ and investigating its drivers have mostly focused on mountainous areas (Huang et al., 2023), urban agglomerations (Wang et al., 2023a), river basins (Sun et al., 2019), and nature reserves (Sallustio et al., 2017), but have ignored ecologically fragile areas which are vulnerable to degradation but difficult to restore, and are largely located in transitional ecotones between two different types of ecosystems (Nguyen and Liou, 2019). Compared to on-fragile areas, these areas are exposed to more pressures from natural and anthropogenic disturbances due to their characteristics of high landscape heterogeneity, large edge-influenced habitats, high sensitivity and low resistance to disturbance (Liu et al., 2021b; Wang et al., 2021b; Shi et al., 2023b). Generally, HQ problems have become increasingly severe in these areas (Zhang et al., 2017; Lv et al., 2019; Wang et al., 2021a). For example, in Alappuzha District, an ecologically fragile area in southern India, a series of environmental problems such as geomorphological degradation, sea level rise, population growth, and anthropogenic damage have been commonly observed (Prasad and Ramesh, 2018). Besides, fragile areas cover more than half of the world’s total land area, playing an important role in ecological security. Thus, these large areas of low habitat quality urgently require restoration of production and function. Therefore, it is of great importance to assess HQ and investigate the driving factors in order to provide a scientific basis for improving HQ in these fragile areas.

Previous HQ assessment methods can be roughly divided into two categories. One was to use data from field surveys and establish an evaluation framework with selected indicators and evaluation standards to assess HQ. This method can directly reflect the ecological conditions of specific sites, but it is time-consuming, labor-intensive, and often fails to consider the spatial interactions among selected ecological features (Goertz, 1964; Ouyang et al., 2001; Xiao et al., 2022). Another one has been to use ecological models to quantitatively assess HQ, such as the MaxEnt (Qi et al., 2011), SoIVES (Wang et al., 2016), HSI (Wang et al., 2009a), InVEST model (Wang et al., 2024a) etc. InVEST model futures the ability to systematically assess ecological and environmental conditions by integrating the impact of threat sources on habitats, allowing it to be applied at multiple scales in various locations all over the world to help solve current and future environmental problems (Pu et al., 2024). Also, it has been proved to be a promising model for HQ assessment in fragile areas.

It is a complex ecological process that spatial HQ patterns and their dynamics have been affected by a combination of natural factors and human activities (Jin et al., 2022). Many studies have shown that natural geographic factors such as soil or topography significantly affected HQ patterns. For example, areas with poor soils tended to have lower HQ (Sun et al., 2019; Jin et al., 2022). Meanwhile, human activities such as population growth, urban expansion and land use change have also been found to significantly affect HQ change, and in some cases become the dominant driver (Bai et al., 2019; Wu et al., 2022; Zhao et al., 2022c). Moreover, these factors of natural disturbance and human activities interact with each other to determine HQ (Li et al., 2019). For example, terrain can not only alter the distribution of temperature and precipitation (Zhao et al., 2023), but also shape human activities to occur mostly at low altitudes (Xu et al., 2020). However, the relative contributions and interactions of different natural and human activity factors to patterns of habitat quality remain unclear, especially in ecologically fragile areas.

The commonly-used methods, such as multiple regression analysis (Yan et al., 2018), correlation analysis (Zhao et al., 2022b) and Geogdetector (Luo et al., 2024), are capable of analyzing the relative contribution of multiple influencing factors to habitat quality, but fail to qualify the interactions among influencing factors. In contrast, Structural Equation Model (SEM) is designed to deal with multiple causal relationships among all variables including explanatory and response variables, and also examine the direct or indirect impacts of explanatory variables on response variables (Grace et al., 2010). As a result, SEM has been widely used to be address multifactor-driven social problems. Recently, it has also been found to be an effective way to quantify interactions among multiple biotic and abiotic factors driving ecological processes (Grace et al., 2016). Therefore, it is essential that SEM be used to explore the combined effects of influencing factors on habitat quality, which will contribute to a better understand of the mechanisms underlying HQ dynamics and ultimately provide valuable information for making policies to improve habitat quality in fragile areas.

The Songnen Plain, located in the temperate semi-humid and semi-arid transitional zone in northeastern China, is an ecologically fragile and intact geographical unit (Wang et al., 2022a). It is a crucial area for China’s grain and livestock production, playing an important role in ensuring national food security and resource supply. Additionally, as an important stopover for migratory birds in Northeast Asia, it is vital for maintaining biodiversity and ecological security within and beyond the region (Yu et al., 2023). In recent decades, the Songnen Plain has faced environmental challenges such as wetland loss, soil salinization, and grassland degradation (Wang et al., 2011). Therefore, it is of great practical importance to systematically study the spatiotemporal changes in habitat quality and identify the driving factors in this region. We performed both the InVEST model and the structural equation model in the Songnen Plain, to (1) assess HQ and analyze the temporal and spatial dynamics in 2000, 2005, 2010, 2015, 2018, and 2020; (2) reveal the impacts of natural and anthropogenic factors and their interactions on the spatial and temporal dynamics of HQ.

## 2 Materials and methods

### 2.1 Study area

**Figure 1:**
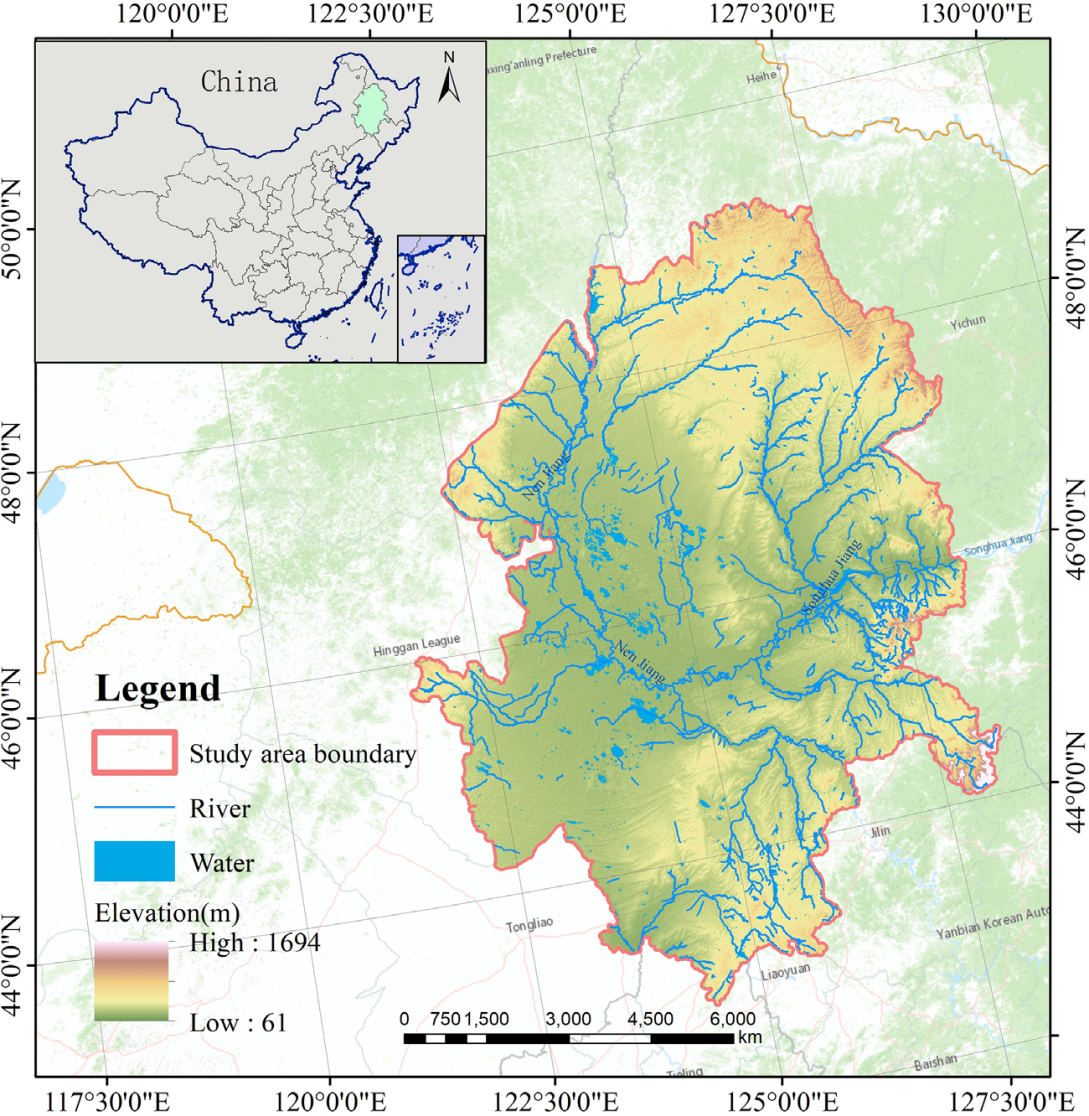
Schematic location of the study area.

The Songnen Plain (E121°38’–128°33’, N 42°49’–49°12’)(**Figure 1**), one of the three major areas with saline-alkaline soils in the world, is situated at the convergence of three transition zones: the Northeast Plain and the Mongolian Plateau, the Semi-Humid Zone and the Semi-Arid Zone, and the Modern Agricultural Zone and the Traditional Animal Husbandry Zone, indicating its fragility in ecological environment (Zhu et al., 2020; Wang et al., 2024b). The study area is characterized by a relatively flat terrain with an average altitude of 180–200 meters. It has a monsoon-influenced temperate continental semi-humid and semi-arid climate with a long cold winter and a short hot summer, and an average annual temperature ranging from 1.6 to 5.0°C. The annual precipitation, ranging from 400 to 600 mm, is concentrated in the summer and decreases from southeast to northwest, while evaporation increases in this direction. This area is abundant in water resources, with the Songhua River, Nen River, Taoer River, Chagan Lake, Dabusu Lake, etc flowing through it. Most parts of this area are covered by black soil and chernozem which are highly suitable for growing crops, making it an important agricultural region in Northeast China. Since the reform and opening up, the Songnen Plain has experienced rapid economic and social development. As of 2018, the total population of the Songnen Plain reached up to approximately 30.5052 million people, with a total GDP of 2,387.826 billion Yuan (Yang and Song, 2021).

### 2.2 Data sources

Land use data: we selected land use data for the years 2000, 2005, 2010, 2015, 2018 and 2020, with a spatial resolution of 30m. We chose this 20-year time scale because it is sufficient to capture the dynamics of the broader-scale system, but land use there changed rapidly and substantially from 2015 to 2020 (Tang et al., 2019). This data set was provided by the Data Center for Resources and Environmental Sciences, Chinese Academy of Sciences (RESDC) (http://www.resdc.cn).

Topographic data: The digital elevation model (DEM) data were derived from the ASTER GDEM 30m digital elevation data product provided by the Geospatial Data Cloud (https://www.gscloud.cn/). We used ArcGIS software to extract two topographic factors of elevation and slope.

Climate data: The High-resolution meteorological raster dataset including “1-km monthly mean temperature dataset for China (1901–2022) (Peng, 2020a)” and “1-km monthly precipitation dataset for China (1901–2022) (Peng, 2020b)” were utilized. These datasets were provided by the National Tibetan Plateau Data Center (http://data.tpdc.ac.cn/) and published by Peng et al (Peng et al., 2017a, b, 2019; Ding and Peng, 2020). Then the average temperature data and annual precipitation data for 2000, 2010, 2018 and 2020 with a spatial resolution of 1km were obtained through preprocessing such as image metric statistics.

Population density data: Population density data for 2000, 2010, 2018 and 2020 were obtained from the World Population Density Map dataset published by WorldPop (https://hub.worldpop.org/) which is the most accurate and reliable long-term series of data available with a spatial resolution of l km.

Nighttime Light: Nighttime light data with a spatial resolution of 1km for 2000, 2010, 2018 and 2020 were obtained from the HARVARD Dataverse (Wu et al., 2021).

Normalized Difference Vegetative Index (NDVI): We utilized NDVI data from the MOD13Q1 product, with a spatial resolution of 250m. We used the Maximum Value Composition to obtain NDVI maximum images for the years 2000, 2010, 2018, and 2020.

Finally, we used resampling techniques to resample all data to a uniform spatial resolution of 1 km.

### 2.3 Methods

#### 2.3.1 Changes in land use 

We employed the land use data of Songnen Plain in 2000, 2005, 2010, 2015, 2018 and 2020 to reveal the area changes of each land use type, and then used the land use transfer matrix to quantitatively represent the conversions between various land use types for each decade from 2000 to 2020 (Yang et al., 2023b). The calculation formula for the land use transition matrix is as follows:


(1) $$T_{ij}=\left(\begin{array}{cccc} T_{11} & T_{12} & \ldots & T_{1n}\\ T_{21} & T_{22} & \ldots & T_{2n}\\ \ldots & \ldots & \ldots & \ldots \\ T_{n1} & T_{n2} & \ldots & T_{nn} \end{array}\right)$$

Where *T_ij_* represents the area that has transitioned from land use type *i* to land use type *j*. *n* is the number of land use types.

#### 2.3.2 Evaluation of habitat quality

We used the HQ module in InVEST to simulate HQ in the years 2000, 2005, 2010, 2015, 2018, and 2020. The InVEST model was developed by Stanford University in collaboration with the World Wide Fund for Nature and other organizations. This model is a modeling system for assessing ecological and economic services of ecosystems to support decision-making for ecosystem management (Wang et al., 2016; Alexander et al., 2018). Based on land use data, the ‘HQ’ module of the InVEST model combines the maximum impact distance, the relative weight of threat factors on habitats, the habitat suitability of each land use type and its sensitivity to threat factors to assess habitat quality at a regional scale. The calculation formula for habitat quality is as follows:


(2) $$Q_{xj}=H_{j}\left(1-\frac{D_{xj}^{z}}{D_{xj}^{z}+k^{z}}\right)$$

Where *Q_xj_* represents the habitat quality of grid *x* within habitat type *j*; *H_j_* is the habitat suitability of habitat type *j*; *z* is a normalization constant; *k* is the half-saturation constant. $D_{xj}$ represents the habitat degradation of grid *x* within habitat type *j*, and its calculation formula is as follows:


(3) $$D_{xj}=\sum_{r=1}^{R}\sum_{y=1}^{Y_{r}}\left(\frac{\omega_{r}}{\sum_{r=1}^{R}\omega_{r}}\right)r_{y}i_{rxy}\beta_{x}S_{jr}$$

Where *y* refers to all grids on the threat grid map for *x*; *Y_r_* refers to a specific set of grids on the threat grid map where the threat factor is *r*; *x* represents the weight of the threat factor; $\beta_{x}$ indicates the accessibility level of grid *x*, with values closer to 1 signifying higher accessibility; $S_{jr}$ is the sensitivity of land cover type *j* to threat factor *r*, with values closer to 1 indicating higher sensitivity. The stress exerted by threat factor *r* in grid *y* on the habitat in grid *x* is represented as $i_{rxy}$ .

When the distance decay effect of threat factor *r* on grid *x* is represented as a linear function, the formula is as follows:


(4) $$i_{rxy}=1-\frac{d_{xy}}{d_{rmax}}$$

When the distance decay effect of threat factor *r* on grid *x* is represented as an exponential function, the formula is as follows:


(5) $$i_{rxy}=\exp\left(-\frac{2.99}{d_{rmax}xy}d_{xy}\right)$$

Where *i_rxy_* represents the impact of grid *y*, where the threat source is *r*, on grid *x*; *d_xy_* is the distance between grid *x* and grid *y*; *d_rmax_* is the maximum range of influence of the threat factor.

First, taking account of knowledge from previous research (Zhao et al., 2022a; Wang et al., 2022b), model guidelines and expert guidance, we determined the model parameters, selected industrial and mining land, arable land, urban land, rural settlements, saline-alkali land and bare land as threat factors (**Table 1**), and identified the maximum impacting distance and relative weight of these 6 threat factors. Then, we identified the habitat suitability and sensitivity of land use types to threat factors (**Table 2**). Second, we ran the HQ module with the above input parameters, producing the simulated maps with HQ value ranging [0,1]. The HQ value closer to 1 represents higher HQ while the HQ value closer to 0 represents lower HQ (Liang et al., 2020; Shi et al., 2023a). Finally, the HQ map was classified into five classes: lowest class [HQ value ∈ (0,0.2)], lower class [HQ value ∈ (0.2,0.4)], medium class [HQ value ∈ (0.4,0.6)], higher class [HQ value ∈ (0.6,0.8)], highest class [HQ value ∈ (0.8,1)].

**Table 1:** Attributes of each threat factor in Songnen Plain.

Threat factor	Threat distance/km	Weights	Type of declining
Industrial and mining land	8.00	0.70	Exponential
Arable land	3.50	0.60	Linear
Urban land	5.00	0.90	Exponential
Rural settlements	4.00	0.60	Exponential
Saline-alkali land	2.50	0.30	Linear
Bare ground	2.50	0.30	Exponential

**Table 2:** Habitat suitability of land use types and sensitivity to threat factors in the Songnen Plain.

Land use type	Habitat suitability	Threat factor					
		Arable land	Urban land	Rural settlements	Industrial and mining area	Saline-alkali land	Bare ground
paddy fields	0.60	0.30	0.50	0.35	0.40	0.50	0.10
dry farmland	0.40	0.60	0.50	0.35	0.40	0.20	0.20
woodland	0.99	0.50	0.85	0.65	0.60	0.60	0.20
shrubland	0.99	0.30	0.70	0.60	0.50	0.60	0.10
sparse woodland	0.99	0.60	0.85	0.65	0.60	0.50	0.30
other woodlands	0.99	0.60	0.85	0.65	0.60	0.20	0.30
high coverage grassland	0.75	0.40	0.60	0.40	0.50	0.25	0.20
medium coverage grass	0.70	0.50	0.70	0.50	0.55	0.30	0.30
low coverage grassland	0.60	0.50	0.80	0.60	0.55	0.30	0.30
river	0.99	0.50	0.90	0.70	0.80	0.25	0.15
lake	0.90	0.50	0.90	0.75	0.80	0.20	0.15
reservoirs	0.90	0.60	0.90	0.75	0.80	0.20	0.15
mudflat	0.70	0.70	0.60	0.60	0.30	0.20	0.30
urban area	0.00	0.00	0.00	0.00	0.00	0.00	0.00
rural settlements	0.00	0.00	0.00	0.00	0.00	0.00	0.00
other construction land	0.10	0.00	0.00	0.00	0.00	0.00	0.00
sandy land	0.00	0.00	0.00	0.00	0.00	0.00	0.00
saline-alkali land	0.50	0.20	0.20	0.15	0.15	0.15	0.10
wetlands	0.65	0.70	0.50	0.20	0.20	0.30	0.30
bare land	0.05	0.00	0.00	0.00	0.00	0.00	0.00
bare rock texture	0.05	0.00	0.00	0.00	0.00	0.00	0.00

Spatial autocorrelation including global autocorrelation and local autocorrelation is used to measure the degree of spatial autocorrelation of geographic object attributes such as HQ value (Choudhary et al., 2020; Huang et al., 2023; Chen et al., 2023a). In this study, the global autocorrelation Moran’s I ranging from -1 to 1 was used to determine the spatial aggregation of HQ value. Moran’s I less than 0, greater than 0, or equal to 0 indicates negative autocorrelation, positive autocorrelation, or no autocorrelation in the spatial distribution, respectively (Sharafatmandrad and Khosravi Mashizi, 2023).

Hotspot analysis is often used to determine, whether,for a geographic feature, there are statistically hotspot areas where grids with high values are significantly clustered or coldspot areas where grids with low values are significantly clustered (Li et al., 2021) (Lane et al., 2018). We used the Getis-Ord Gi* index to identify hot/cold spot areas of HQ.

#### 2.3.3 Impacts of influencing factors on habitat quality

We used structural equation modelling (SEM) to simulate the relationships between HQ and the influencing factors. As a powerful statistical method for multivariate analysis, SEM is distinctive by the combination of causal and measurement models, the consideration of interactions among multiple influencing variables, and the assessment of the impact of potential variables (He et al., 2020). It can be tested by fitness to improve its reliability and stability (Ye et al., 2022; Wang et al., 2023b). In the SEM simulation, a conceptual model was first constructed, considering both observed variables and latent variables. The observed variables can be directly observed or measured, while latent variables cannot be directly observed. We tested and optimized the constructed concept model according to the fitness, and then obtained an optimized concept model map explaining the relationships among all explanatory and response variables (Jöreskog, 1970). The formula for the Structural Equation Model (SEM) is as follows:


(6) Y=Λy η+ϵ

Where *Y* represents the observed variable; *η* represents the latent variable; Λy is the factor loading matrix; and ϵ epsilonϵ represents the measurement error.


(7) η=Bη+Γξ+ζ

Where *B* is the path coefficient matrix between endogenous latent variables; Γ is the path coefficient matrix from exogenous latent variables to endogenous latent variables; and *ζ* represents the structural error.

**Figure 2:**
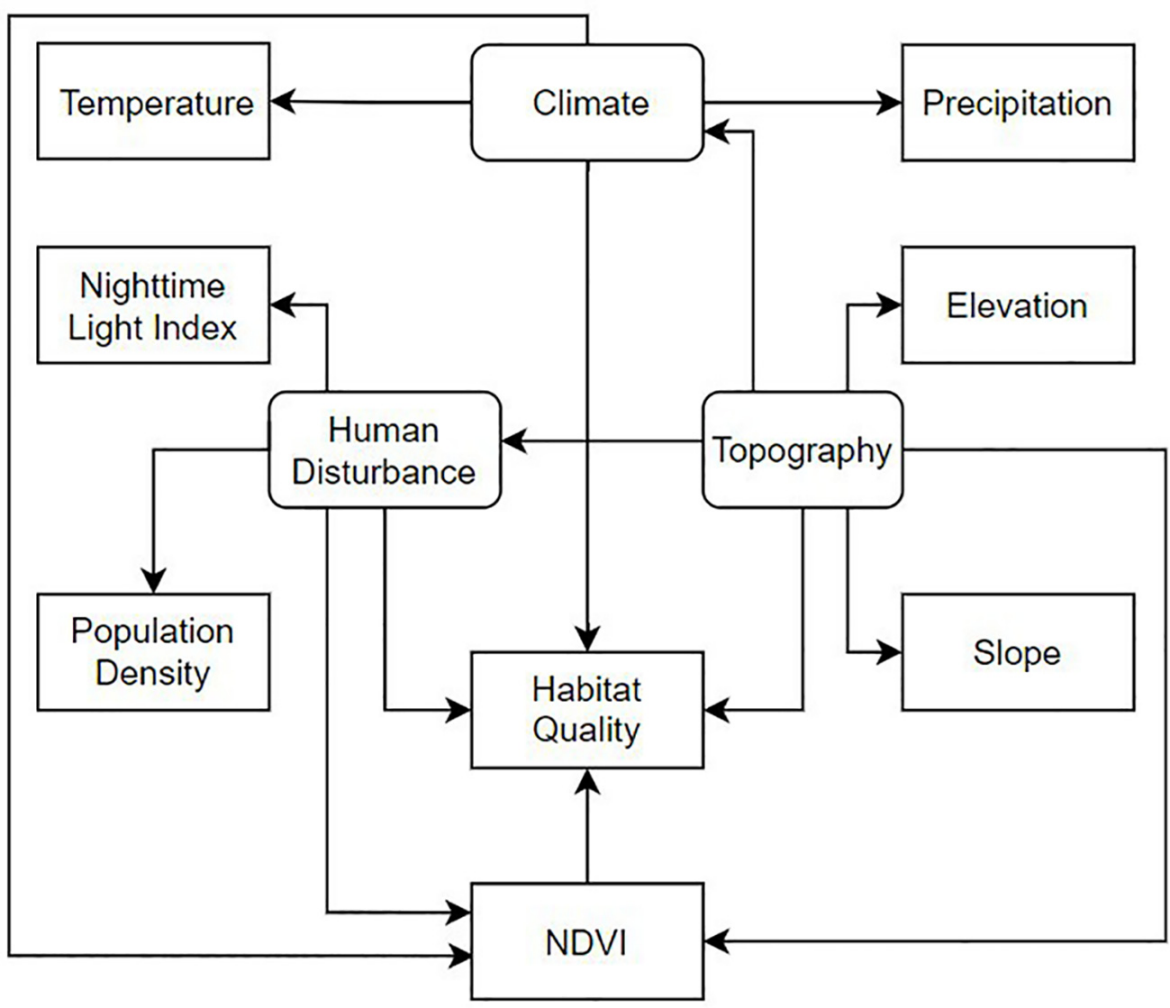
Conceptual model of influencing factors of HQ in the Songnen Plain, 2000–2020. Rounded rectangles are latent variables; rectangles are observed variables; Directional paths (one-way arrows) indicate causal relationships between two variables.

HQ is usually influenced by natural and anthropogenic factors (Fritz et al., 2018). Taking into account the natural ecological environment and the demands of socio-economic development of the Songnen Plain (Jasperson et al., 2018), according to previous studies, data availability, and consideration of the regional characteristics of the Songnen Plain (Rahimi et al., 2020), we selected a total of five natural elements and two social factors as factors influencing HQ, including elevation, slope, annual precipitation, average annual temperature, Normalized Difference Vegetative Index(NDVI), population density and nighttime lighting, and established a conceptual model of the spatial-temporal distribution factors influencing HQ (**Figure 2**). NDVI is widely used as a measure of vegetation health, and nighttime light intensity is a common indicator of the intensity of human activity. Topography, human disturbance and climatic conditions were the latent variables, and each kind of latent variable consisted of two observational variables. Topography included elevation and slope, human disturbance included night-time lighting and population density, and climatic conditions included annual precipitation and annual mean temperature.

First, we tested eight variables (seven explanatory variables and one response variable) for normality. To satisfy the normality assumption, we performed Johnson transformations on the variables that did not meet the normality criteria and then standardized the variables. Second, to identify the best fitting model, four indicators were selected to analyze the effectiveness of model fitting, including Comparative Fit Index (CFI), Goodness of Fit Index (GFI), Root Mean Square Error of Approximation (RMSEA), and Standardized Residual Mean Root (SRMR). Generally, If the model fitness meets the requirements of CFI > 0.90, GFI > 0.90, RMSEA < 0.06 and SRMR < 0.05 (Team, 2013), the model results were expected to be presented in a path diagram with paths marked by a corresponding standardized Regression Coefficient (r) indicating the strength of the causal effect between the variables at the two ends of that path. The path with a larger coefficient means a stronger effect. If a variable points directly to the HQ, the effect of that variable was direct, while, if a variable does not point directly to the HQ, the effect of that variable was indirect. The product of the coefficients of all paths from that variable to the response variable indicated the magnitude of that effect. The magnitude of direct and indirect impacts of the variables was the total impact.

## 3 Result

### 3.1 Changes in land use

**Figure 3:**
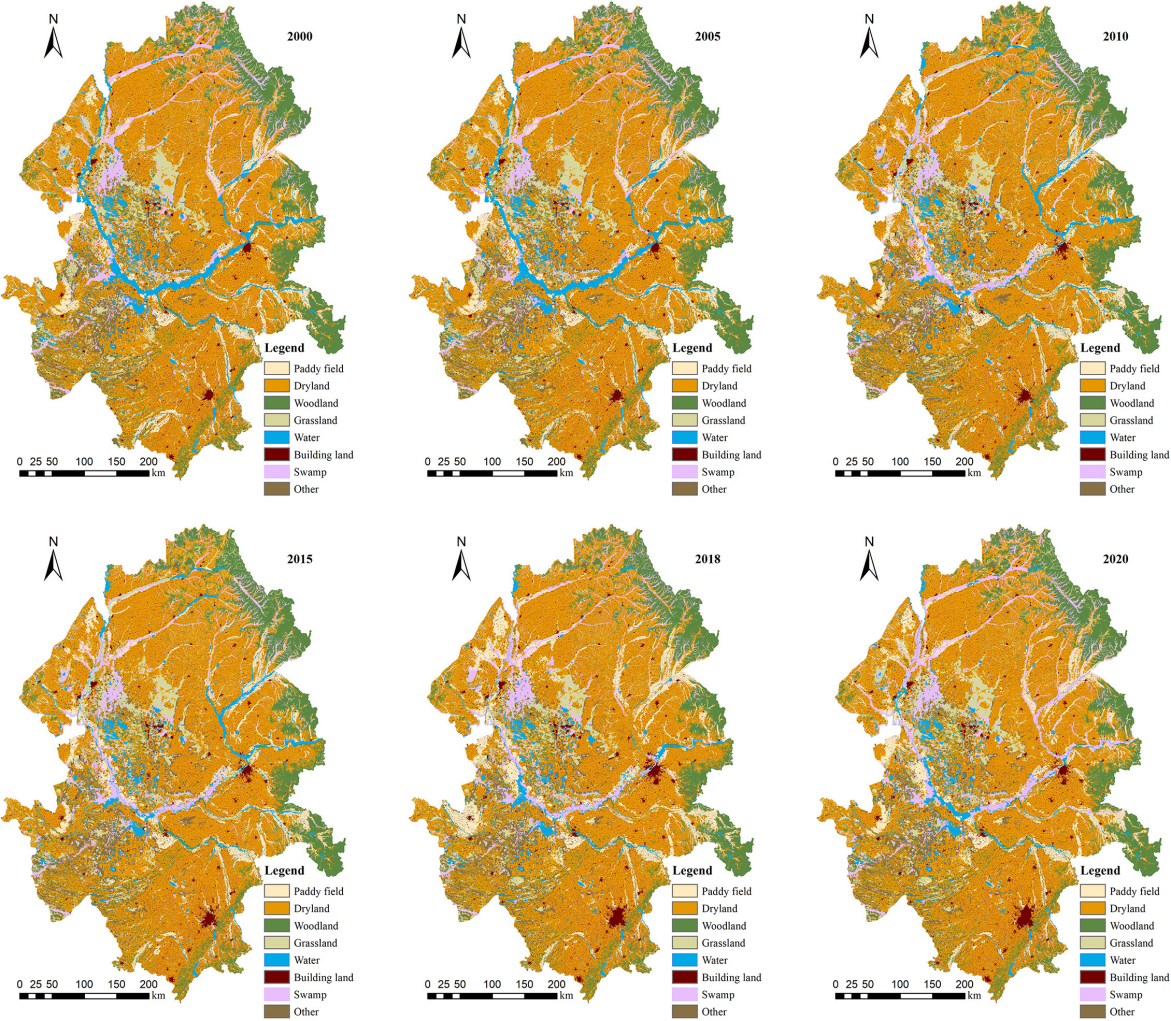
Spatial distribution of land use in the Songnen Plain, 2000–2020.

**Figure 4:**
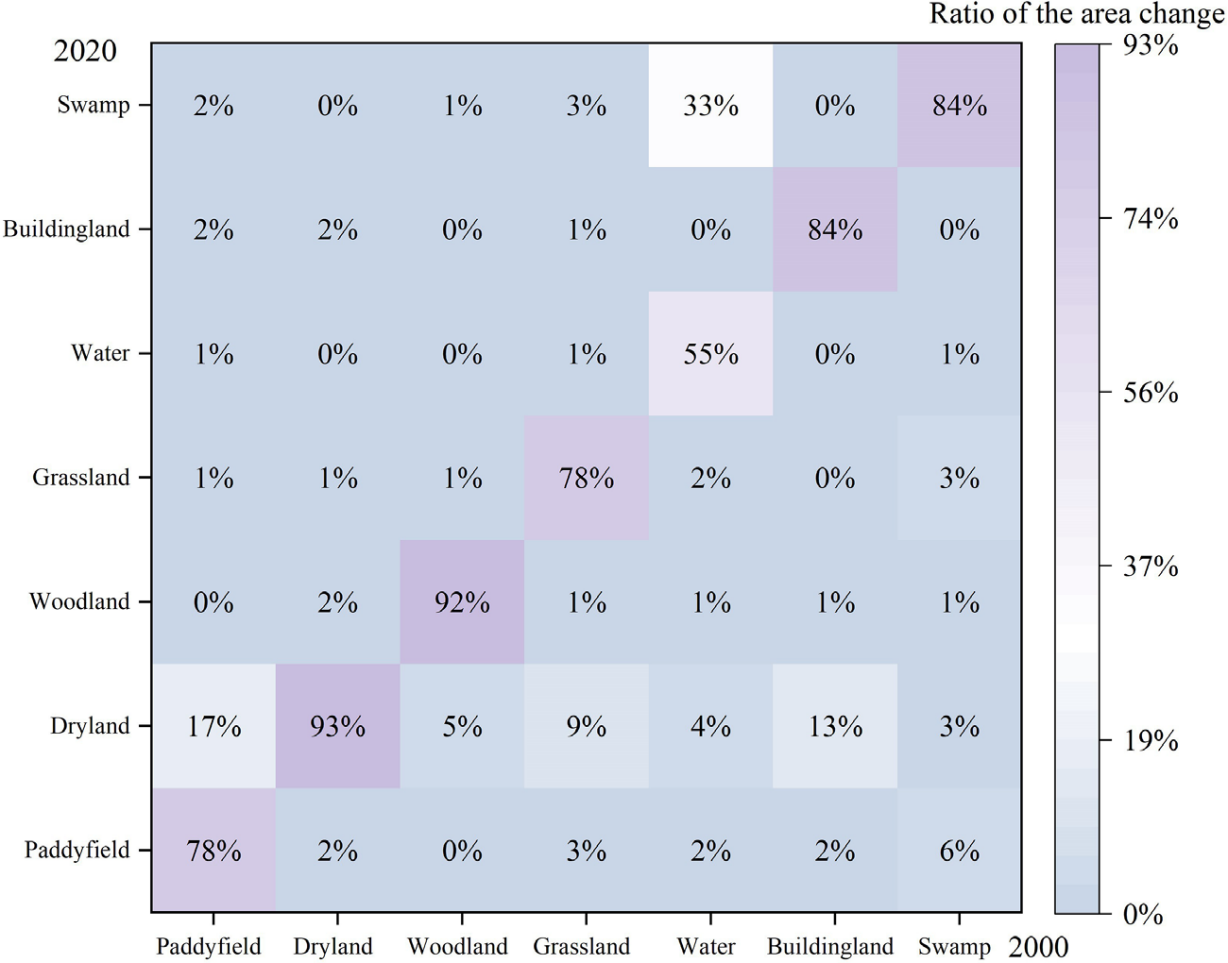
Transition matrix of land use in the Songnen Plain, 2000–2020.

In the six periods of 2000, 2005, 2010, 2015, 2018, and 2020, land use type with the largest area percentage in the Songnen Plain was arable land, followed by woodland (**Table 3**). Arable land was mainly concentrated in the central and western parts, while woodland was in the mountainous and hilly areas in the eastern part (**Figure 3**). During 2000–2018 (**Table 3**), the arable land dominating the Songnen Plain gradually increased in size from 59.10% to 61.68%. In particular, paddy land increased from 6.43% in 2000 to 8.59% in 2018, and dry farmland increased from 52.67% in 2000 to 53.09% in 2018. The percentage of forested land decreased from 12.14% to 11.88%. Grasslands, swamps and waters had also decreased gradually. During the period 2018–2020, the area of arable land decreased by 1.78%, the area of woodland increased from 11.88% to 12.3%, and the area of grassland and swamps also increases to 7.42%. The area of building land was reduced to 4.9%. The transformation matrix (**Figure 4**) showed that during the period 2000–2020, the most obvious change in paddy land was the conversion to dry farmland with a conversion rate of 17%, followed by the conversion to building land, with a conversion rate of 2%. The largest change was from woodland to dry farmland, with a conversion rate of 5%. Grasslands and swamps were also converted mainly to dry farmland, with conversion rates of 9% and 3%. Overall, land-use change in 2000–2020 was characterized by a continuous conversion of woodland, grassland and swamp to arable land.

**Table 3:** Percentage area (%) of land use types in Songnen Plain, 2000–2020.

Land use type	2000	2005	2010	2015	2018	2020
paddy fields	6.43	6.23	5.98	6.37	8.59	6.95
dry farmland	52.67	53.27	53.92	53.67	53.09	52.56
woodland	12.14	12.11	11.95	11.93	11.88	12.30
grassland	7.96	7.95	8.26	8.19	6.57	7.42
waters	4.90	4.72	3.92	3.91	3.30	3.19
building land	4.33	4.38	4.68	4.77	5.18	4.90
swamp	6.13	5.89	5.69	5.62	6.22	7.42
other	5.43	5.44	5.60	5.54	5.17	5.26

### 3.2 Changes in habitat quality

#### 3.2.1 Changes in habitat quality pattern

**Figure 5:**
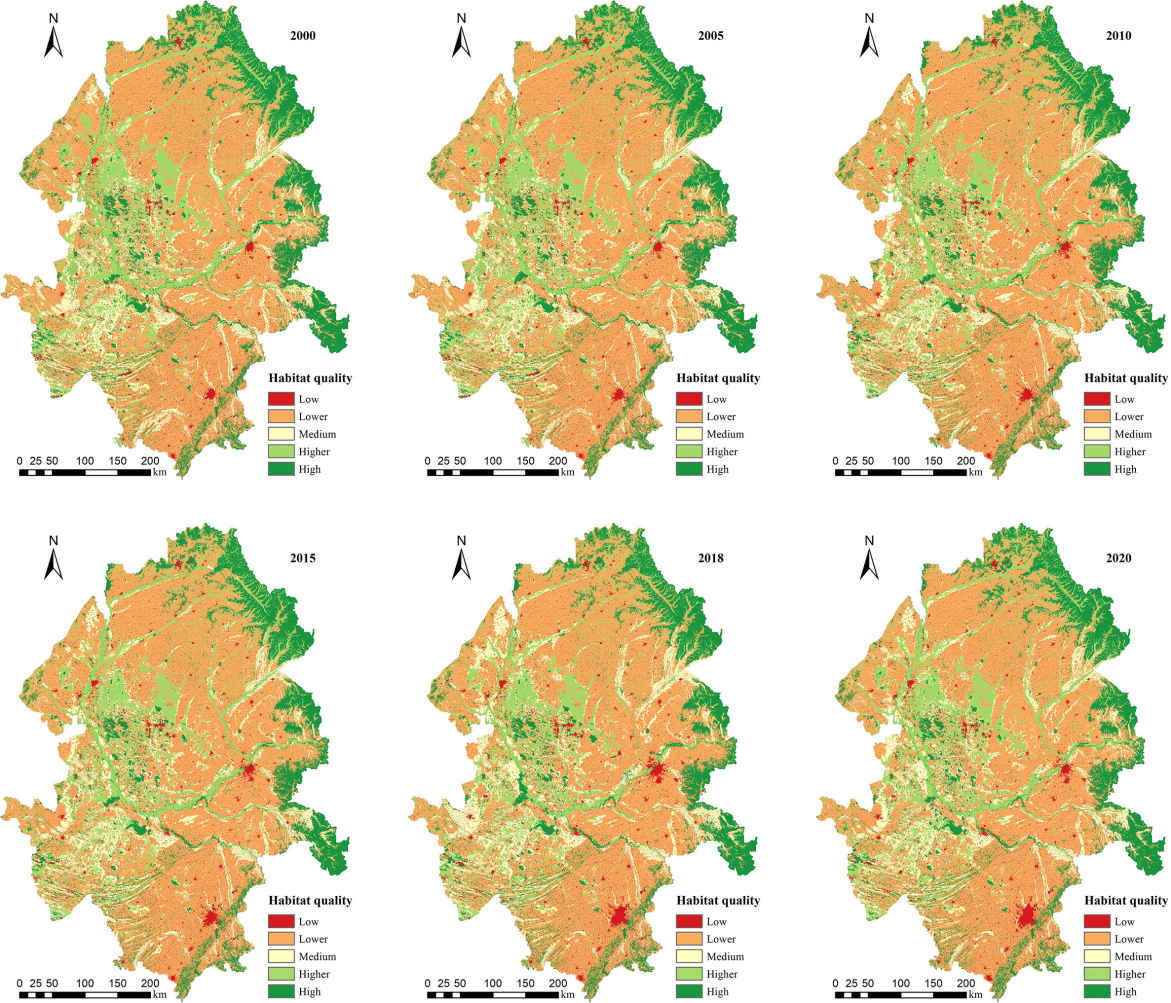
Distribution of HQ grade in the Songnen Plain, 2000–2020.

The results of the InVEST model showed that the overall level of HQ was lower in all six periods (**Figure 5**). Meanwhile, the spatial patterns of HQ were highly heterogeneous, as indicated by the trend of ‘high HQ in the east – low HQ in the center – high HQ in the west’. Areas of high HQ were concentrated along the eastern edge and scattered in small patches in the center. These highest-value areas mainly fall in ecological lands with high vegetation cover, rich biodiversity and less human activities, such as woodlands and swamps. The lower HQ areas were mainly located in the central farmland areas with intensive agricultural activity. The lowest HQ areas were concentrated in these middle areas which are densely populated and highly industrialized, leading to more fragile ecological environments.

The average values of HQ index of Songnen Plain in 2000, 2005, 2008, 2010, 2015, 2018 and 2020 were 0.534, 0.532, 0.532, 0.528, 0.527, 0.52 and 0.530, respectively. HQ showed a strong continuous downward trend due to the obvious expansion of arable land and building land during 2000–2018 and appeared to improve after 2018.

**Table 4** showed that more than 50% of the area in the Songnen Plain had lower HQ in all six periods. The percentage of the lowest-grade HQ area gradually increased from 4.50% to 5.36% and the lower-grade HQ area increased from 52.67% to 53.92% during 2000–2018. The percentage of the highest-grade HQ area decreased, with the percentage of the highest-grade area decreasing from 16.22% to 15.00% and the percentage of the higher-grade area decreasing from 14.67% to 14.59%. The expansion of lowest and lower HQ areas and the contraction of the high and higher HQ areas indicated a decrease in HQ and gradual ecological degradation. During 2018–2020, HQ improved, with the percentage of the highest-grade area increasing to 15.32% and the percentage of the higher-grade area increasing to 14.72%. This positive trend in HQ illustrated that the ecological environment of the Songnen Plain has improved significantly in recent years.

**Table 4:** The percentage of HQ grade area in the Songnen Plain, 2000–2020 (%).

year grade	2000	2005	2008	2010	2015	2018	2020
Lowest	4.50	4.55	4.59	4.86	4.95	5.36	5.09
lower	52.67	53.27	53.09	53.92	53.67	53.09	52.55
medium	11.94	11.75	11.88	11.71	12.04	13.90	12.32
higher	14.67	14.50	14.57	14.36	14.33	14.59	14.72
highest	16.22	15.93	15.88	15.16	15.00	13.06	15.32

#### 3.2.2 Changes in hotspot pattern of habitat quality

**Figure 6:**
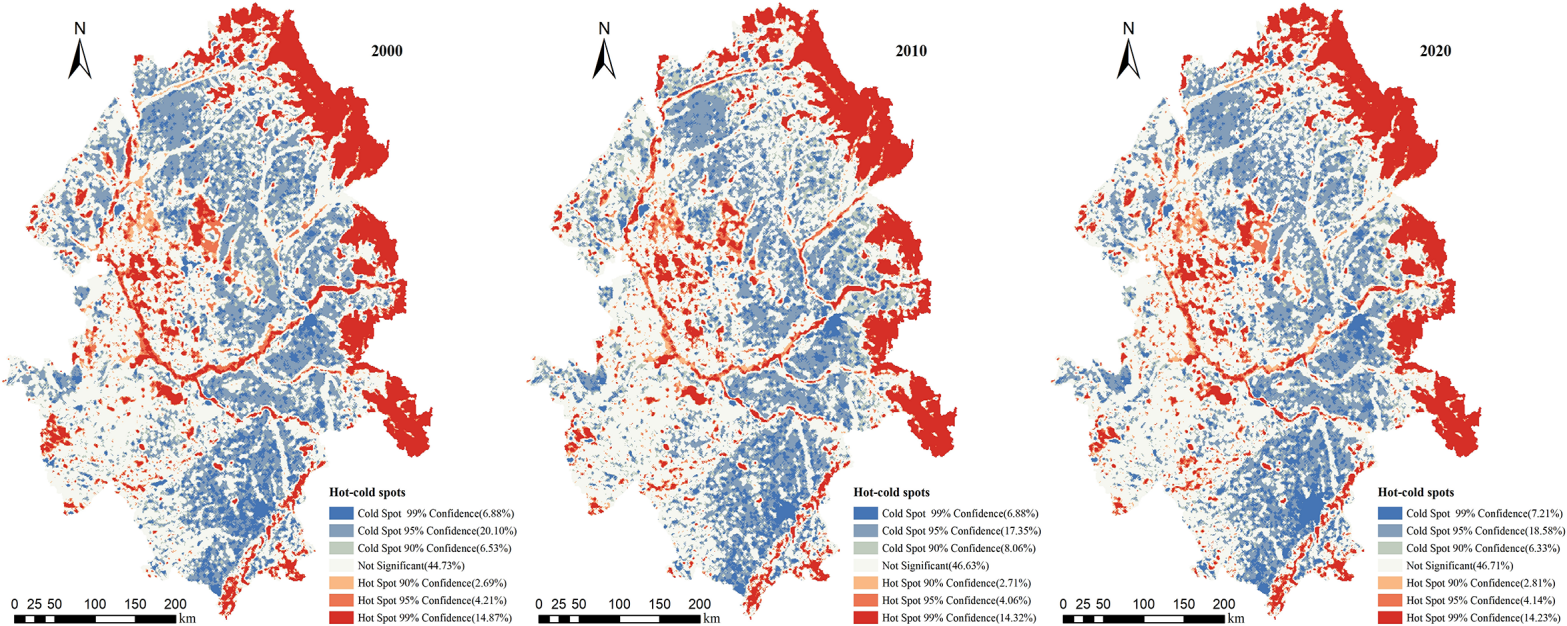
Hot/cold spot analysis of HQ in the Songnen Plain, 2000–2020.

The Moran’s I of HQ in the Songnen Plain in 2000, 2010, and 2020 were all greater than 0, indicating a certain degree of spatial clustering distribution of HQ. The values of Moran’s I in 2000, 2010, and 2020 were 0.516, 0.507. and 0.508, respectively, showing a slight decrease followed by an increase in spatial clustering of HQ. **Table 5** showed a decreasing trend in hotspot areas and an increasing trend in coldspot areas in the Songnen Plain from 2000 to 2020. The hotspot analyses of Getis-Ord G* (**Figure 6**) revealed that the spatial distribution of high and low HQ values remained relatively consistent from 2000 to 2020. The hotspots areas were largely located in the eastern part of the Songnen Plain, which was characterized by high elevation, dense vegetation cover, and good HQ. Hotspots were also found in the vicinity of Songhua River, Nenjiang River and other rivers, where there was less human disturbance occurred. Coldspot areas were mainly distributed in the central area of Songnen Plain. Building land and arable land were the main land use types in the central area which were greatly affected by human activities.

**Table 5:** Percentage area of hot/cold spots of HQ in the Songnen Plain, 2000–2020 (%).

HQ coldspots	2000	2010	2020
Coldspot – 99% Confidence	6.88	6.88	7.21
Coldspot – 95% Confidence	20.10	17.35	18.58
Coldspot – 90% Confidence	6.53	8.06	6.33
Not Significant	44.73	46.63	46.71
Hotspot – 90% Confidence	2.69	2.71	2.81
Hotspot – 95% Confidence	4.21	4.06	4.14
Hotspot – 99% Confidence	14.87	14.32	14.23

#### 3.2.3 Degradation of habitat quality

**Figure 7:**
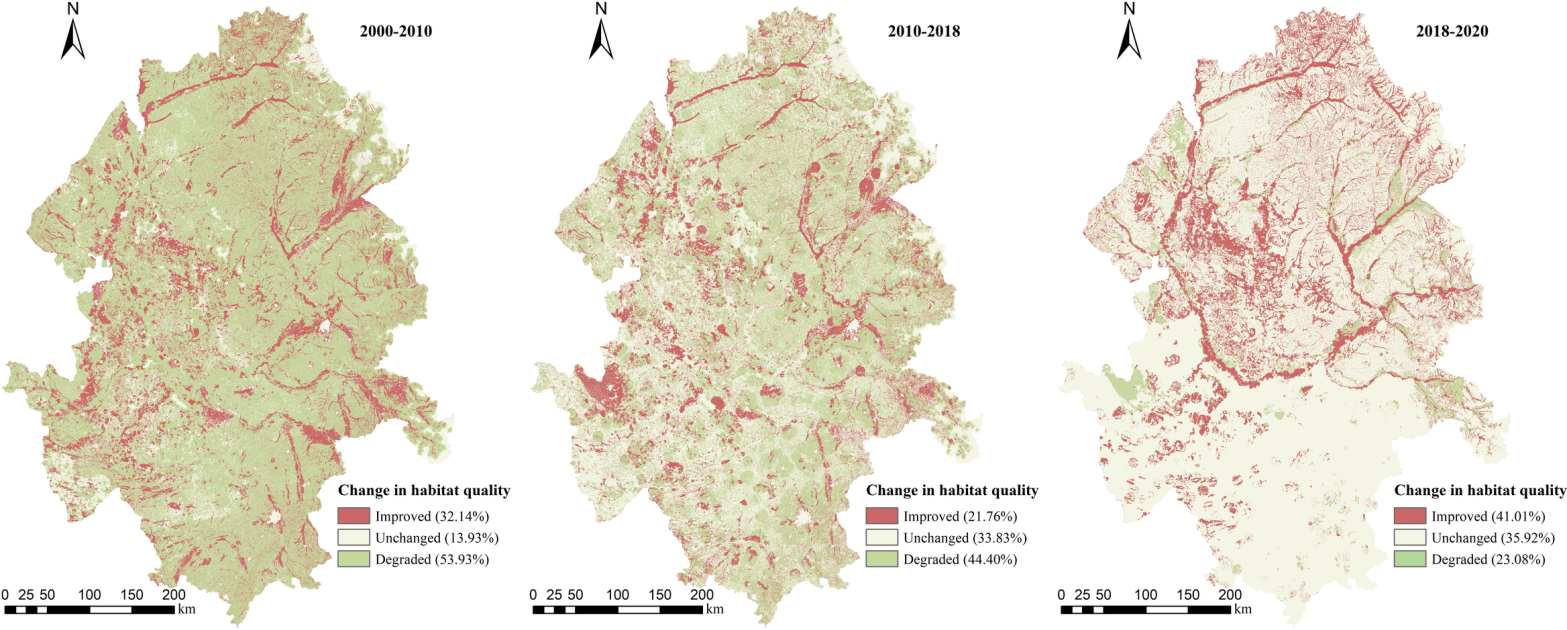
Distribution of HQ change in the Songnen Plain, 2000–2020.

The distribution of habitat quality changes is shown in **Figure 7**. From 2000 to 2010, the percentage of degraded HQ areas in the Songnen Plain was as high as 53.93% (**Table 6**), and these areas were widely distributed. During this period, particularly from 2005 to 2010, the implementation of the Northeast Revitalization Strategy led to rapid economic development. Consequently, HQ degraded due to frequent industrial and agricultural activities. From 2010 to 2018, the degraded areas still dominated with 44.40% but showed a slight decrease compared to the period from 2000 to 2010. The area with unchanged HQ increased accounted for 33.83% (**Table 6**), indicating the implementation of policies considering both economic development and environmental protection. In 2018–2020, the areas with improved HQ were widely distributed, reaching up to 41.01% of Songnen plain. The percentage of degraded areas decreased to 23.08%, demonstrating that ecological environment protection and restoration practices progressively enhanced the habitat quality.

**Table 6:** Percentage of area (%) of change in HQ in the Songnen Plain, 2000–2020.

Changing trends of HQ	2000–2010	2010–2018	2018–2020
Improved	32.14	21.76	41.01
Unchanged	53.93	44.40	23.08
Degrade	13.93	33.83	35.92

### 3.3 Impacts of influencing factors on habitat quality

The SEM model of HQ influencing factors in the Songnen Plain met the required goodness-of-fit indicators (**Table 7**), 2000 (CFI=0.99, GFI=0.996,RMSEA=0.038,SRMR=0.012), 2010 (CFI=0.965, GFI=0.993,RMSEA=0.031,SRMR=0.031), 2018 (CFI=0.952, GFI=0.997,RMSEA= 0.044,SRMR=0.028), and 2020 (CFI=0.941, GFI=0.992,RMSEA=0.021,SRMR=0.018), indicating that fitness met the requirements.

**Table 7:** Fitness test for structural equation fitting.

Fitting index	Adaptation standards	2000	2010	2018	2020
CFI	>0.90	0.991	0.965	0.952	0.941
GFI	>0.90	0.996	0.993	0.997	0.992
RMSEA	<0.06	0.038	0.031	0.044	0.021
SRMR	<0.05	0.012	0.031	0.028	0.018

**Figure 8:**
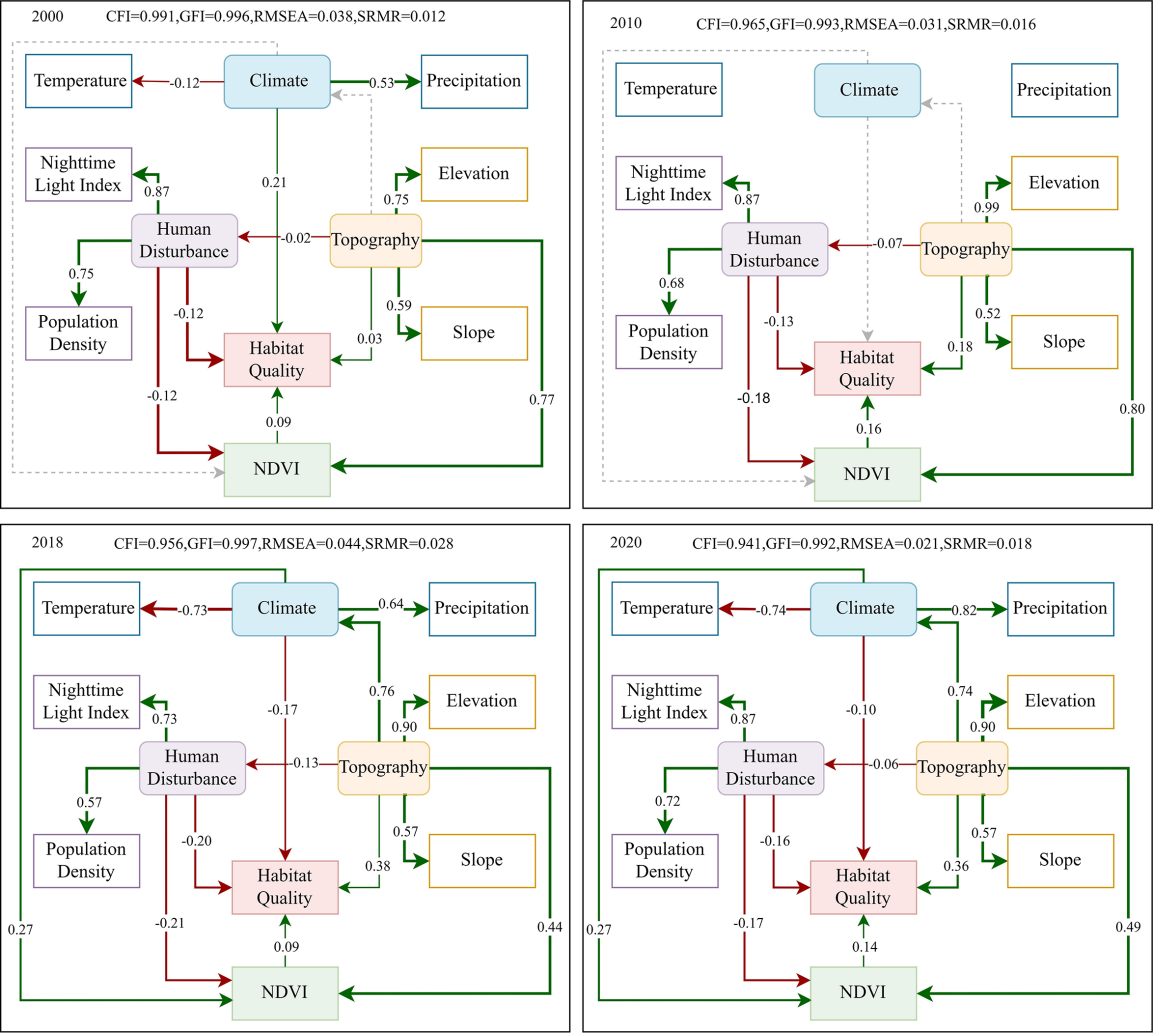
Structural equation model for the comprehensive effect of influencing factors on HQ. Green lines indicate positive impacts; red lines indicate negative impacts; solid lines indicate paths that passed the significance test (p<0.05); dashed lines indicate paths that are not significant; line widths indicate impact size

SEM of HQ influencing factors (**Figure 8**) showed that in 2000 (**Figure 8A**), climate, topography, NDVI, and human disturbance combined to influence HQ. Climate had a strong direct positive influence on HQ with a coefficient of 0.21(r=0.21). Specifically, temperature had a negative effect on HQ, while precipitation had a positive effect, indicating that lower temperature, or higher precipitation produced higher HQ. NDVI had a small positive effect on HQ with a coefficient of 0.09. Topography also had an important effect on HQ in three pathways: 1) directly affecting HQ with an impact coefficient of 0.03, 2) indirectly affecting HQ through NDVI with an impact coefficient of 0.069, and 3) indirectly positively affecting HQ through limiting human disturbances with an impact coefficient of 0.002. The total effect of topography on HQ was 0.1. Human disturbance had a negative impact on HQ with an overall impact coefficient of −0.13. This included a direct effect (r=−0.12) and an indirect effect by influencing NDVI (r=−0.01).

In 2010 SEM (**Figure 8B**), there was no pathway between climate factors and HQ, indicating that climate did not have a significant effect on HQ. Topography was the most important driver of HQ, with a total effect coefficient of 0.32. This included a positive direct effect (r = 0.18), as well as indirect effects through influencing NDVI (r = 0.13) and indirect effects through influencing human disturbance (r = 0.01). The total impacts of human disturbance on HQ were negative, including negative direct impacts (r=−0.13) and negative indirect impacts through NDVI (r=0.03). The total impact coefficient was -0.16, larger than 0.03 compared to 2000 (r=−0.13).

The 2018 SEM showed (**Figure 8C**) that topographic factors, as the strongest factor, had a strong overall positive effect (r=0.32), including a positive direct effect (r=0.38), a positive indirect effect through NDVI and human disturbance (r=0.07), and a negative indirect effect through climate (r=−0.13). The positive effect of topography on NDVI (r=0.44) indicated that areas with higher elevations and greater slopes had higher NDVI and tended to have higher HQ. Topography had a negative effect on human disturbance (r=−0.13), suggesting that areas with low elevation, low slope, and low topographic relief consisted of large urban area and more human activity. Human disturbance had the strongest negative effect on HQ, with a total impact coefficient of −0.23, including a strong negative direct effect on HQ (r=−0.20) and a negative indirect effect through NDVI (r=−0.03). Climate impacts on HQ were negative with a total impact coefficient of −0.15, including negative direct impacts with an impact coefficient of −0.17, and positive indirect impacts through NDVI (r=0.02).

The 2020 SEM showed (**Figure 8D**) that topography continued to be the strongest influence on HQ, with a total effect coefficient of 0.36. Topography had a very strong positive direct effect on HQ, with an effect coefficient of 0.36. Topography also had a positive indirect effect on HQ through its influence on NDVI and human disturbance (r=0.07), and a negative indirect effect on HQ through its influence on air temperature (r=−0.07). Human disturbance appeared to have the strongest negative influence on HQ, with an overall impact coefficient of −0.18, consisting of a strong negative direct effect on HQ (r=−0.16) and a weak negative indirect effect through NDVI (r=−0.02). Climate had a negative impact on HQ with a total impact coefficient of −0.12. This included a direct impact coefficient of −0.1 and an indirect impact coefficient of −0.02. Temperature had a positive impact on HQ, while precipitation had a negative impact. NDVI effected HQ with the coefficient of 0.14.

## 4 Discussion

### 4.1 Changes in habitat quality

The HQ of the Songnen Plain from 2000 to 2020 was low, which indicated the fragility of the ecological environment. Our results are exactly supported by Chen et al (Chen et al., 2020) and Yang who found that HQ was lower in the Songnen Plain during this period (Yang et al., 2021). Our study also found that the distribution of HQ in the Songnen Plain was spatially heterogeneous, showing a ‘high-low-higher’ pattern from east to west. In the eastern part of the region, large-area woodland, rich biodiversity, and minimal human disturbance resulted in relatively high HQ. In the flooded central and western regions, original land use types such as swamps and grasslands have been exploited by human activities to meet the demands of social development, resulting in low HQ. Our results indicated that the average value of HQ in the Songnen Plain generally decreased from 2000 to 2018 but increased from 2018 to 2020, which is similar to the trend in other studies. For example, Dai et al. found that HQ decreased due to landscape fragmentation in Northeast China from 1990 to 2010 (Dai et al., 2018). Chen et al. found that the HQ of Heilongjiang and Jilin provinces was lower in 2020 than in 2010 (Chen et al., 2023a).

Our study found that the area of lowest-grade and lower-grade HQ in the Songnen Plain continued to expand, while the area of highest-grade and higher-grade HQ decreased between 2000 and 2018, which is consistent with other existing findings (Chi et al., 2023; Li et al., 2023a). During this period, the Songnen Plain, as an important commercial grain base in China, was largely reclaimed from ecologically functional land use including grasslands and wetlands, to farmland to meet the growing demand for food. Although the implementation of ecological projects such as ‘Grain for Green’ maintained the HQ to some extent, these reclamation practices aimed at short-term economic benefits led to the degradation of the HQ in less economically developed areas (Wang et al., 2015; Jin et al., 2022).

Our study showed that during 2018–2020, the area of high-grade HQ increased to 15.32% and the area of higher-grade HQ increased to 14.72%, illustrating an upward trend in HQ. This was partly due to the restoration of damaged ecosystems as a result of the enforcement of environmental protection and restoration policies (Jasperson et al., 2018; Mao et al., 2021). That is, the national government has implemented several ecological conservation policies, such as the Natural Forest Conservation Project, the Grain to Green Project, and the Land Salinity/Sodicity Amelioration Project since 2000, and also proposed the goal of “accelerating the progress of ecological civilization” in 2015, which ultimately reversed the trend of habitat quality degradation around 2018 (Zedler, 2003). Through scientific and rational management, and the implementation of ecological protection policies, damaged ecosystems have been restored, leading to a significant improvement in habitat quality. Similarly, previous studies have shown that ecological protection projects and ecological restoration projects, such as the establishment of efficient ecological economic zones and ecological counties, have restored ecosystems to some extent and slowed down the decline of habitat quality (Sun et al., 2022; Zheng et al., 2022).

### 4.2 Impacts of influencing factors on habitat quality

The 2000 SEM showed that climate had the greatest influence on HQ, with temperature having a negative effect and precipitation having a positive effect. Since 1980, the annual temperature in the Songnen Plain had increased at a rate of more than 0.44°C per decade (Wang et al., 2011), which was found to cause the loss of swamp and grassland areas and subsequently the decrease in HQ (Wang et al., 2009b). In addition, Yang et al. found that Northeast China experienced eight months of severe drought from 2000 to 2001 (Yang et al., 2023a). During the drought period, a small amount of precipitation could promote HQ (Liu et al., 2023), illustrating the significant influence of precipitation, which supported our findings. Precipitation affects vegetation growth and distribution by influencing soil moisture and vegetation sensitivity to temperature, which in turn impacts habitat quality (Du et al., 2019). The effects of temperature and precipitation on HQ varied across years. There was a negative effect of temperature and a positive effect of precipitation in 2000, while, the directions of these effects were completely reversed in 2018 and 2020. Zheng and Li showed that the Songnen Plain experienced a wet period from 2018 to 2020 with a maximus number of extreme rainfall events in 2019 (Zheng and Li, 2022). Excessive precipitation in this wet year exacerbated soil erosion, negatively impacting HQ (Liu et al., 2022). In addition, our results indicated that precipitation and temperature affect HQ with varying intensities. That is, intensity of the positive impact of precipitation was higher than that of the negative impact of temperature in 2000, the intensity of the positive impact of temperature outweighed that of the negative impact of precipitation in 2018, and the intensity of the negative impact of precipitation was higher than the intensity of the negative impact of temperature in 2020. However, some studies demonstrated that precipitation had a greater influence on HQ than temperature (Fritz et al., 2018). This different conclusion from ours could be attributed to the different ecological spatiotemporal characteristics between the study areas.

Our study found that topography significantly influences HQ in the SEM during four years: 2000, 2010, 2018, and 2020, which is supported by some previous research indicating that HQ varies across topographies (Feng and Lei, 2023; Li et al., 2023a, b). HQ was higher in mountainous regions where forests were well-preserved, while HQ was lower in both hilly areas with intense agricultural activities and in plains with high population (Feng and Lei, 2023; Li et al., 2023a, b). In addition, we found that topography indirectly impacts HQ. Previous studies had shown that topography affect HQ by influencing climate, NDVI, and human activities to varying extents (Chen et al., 2023b; Shi et al., 2023b). For instance, Yang et al. reported that elevation and slope direction influence temperature and precipitation, finally determining forest distribution in the Qilian Mountains of northwestern China (Yang et al., 2018). Liu et al. demonstrated that elevation affects NDVI averages, and impacted vegetation distribution (Liu et al., 2021a). Chen et al. showed that lower- and mid-elevation areas were more vulnerable to human disturbance (Chen et al., 2023b). All of these studies highlighted the significant impact of topography on HQ (Xiao et al., 2022).

Our results showed that human activities involving nighttime light and population had the strongest negative impact on regional HQ. Nighttime light intensity and population density were found to reflect urban expansion and industrial agricultural activities. Other studies had found that human disturbance significantly affects HQ, e.g. Bai et al. found that human disturbance exacerbated the degradation of HQ in Changchun City (Bai et al., 2019), and Zhao et al. found a significant positive correlation between the intensity of human activities and HQ (Zhao et al., 2022c). Our study also showed that human disturbance had a negative effect on NDVI. Zhou et al. studied the historical dynamics of vegetation in China and found that human activities affected NDVI (Zhou et al., 2020). Additionally, our study demonstrated that the use of nighttime light data was a feasible solution to assess the effect of human activities on HQ, which was supported by Zhao et al (Zhao et al., 2022c).

Our results suggest that the overall effect of natural environmental factors on HQ in the Songnen Plain exceeds the total effect of human disturbance, but the effect of human disturbance on habitat quality is greater than that of natural environmental factors in some small areas due to the heterogeneity in landscape characters, and the intensity of the effect of each driver varies over time. Other studies conducted at different temporal and spatial scales have reached similar conclusions in other ecologically fragile areas, e.g., Mengist found that anthropogenic disturbances had affected HQ in a forested biosphere reserve in southwest Ethiopia (Mengist et al., 2021). In another fragile area, Ebinur Lake Basin (Xinjiang Province, China), land use changes reduced by human disturbance were found to affect HQ (Wei et al., 2022). Meanwhile, similar findings have been found in non-fragile. For example, Chen et a concluded that natural environmental factors determine the spatial pattern of HQ in the central region of the Yangtze River Delta (Chen et al., 2023c), and Wu et al. found that HQ was mainly influenced by natural factors in the Guangdong-Hong Kong-Macao Greater Bay Area from 2000 to 2020 (Wu et al., 2024).

### 4.3 Management implications

In this study, we obtained qualified evidence that the habitat quality in the fragile Songnen Plain is subject to multiple impacts from natural and anthropogenic factors, which has implications for ecological management in this study area and other similar fragile ecological zones. First, the eastern part of the Songnen Plain covered by a large area of forest with high habitat quality should be protected by management practices such as afforestation and fire prevention to maintain the habitat stability quality. Second, ecological projects such as Saline-alkali Land Restoration and Grain for Green need to be continuously implemented to improve the low habitat quality in the central and western parts of the Songnen Plain. Third, SEM results showed that precipitation and temperature have significant effects on habitat quality. To mitigate the negative threats to habitat quality from extreme weather events such as drought and flood, long-term monitoring of climate change, utilization of existing water infrastructure, and improved water management are needed (Sun et al., 2021). Fourth, the negative effects of human activities on habitat quality should not be ignored. In urban and rural development and construction, the landscape pattern can be optimized from an ecological protection perspective (Pu et al., 2024) to improve landscape functions. In sum, all those practices will help to balance economic growth and ecological land demand to improve the habitat quality of the entire region.

## 5 Conclusions

HQ in the Songnen Plain was low from 2000 to 2020, with significant heterogeneity in spatial distribution. HQ continued to decline from 2000 to 2018, and then recovered slightly from 2018 to 2020. The overall impact of natural factors on HQ in the Songnen Plain was stronger than that of human disturbance, and the intensity of the effects of the natural environment and human disturbance on HQ varied over time. Complex interactions between the natural environment and human disturbance were also important in influencing HQ. Our methodology and results have imporatn implications for ecological conservation in the Songnen Plain and provide insights for studying HO in other regions. Our study highlighted that HQ could be improved by regulating development and management practices, and offered strong evidence for demonstrating relationships between influencing factors and HQ in ecologically fragile areas. 

## Data Availability

The original contributions presented in the study are included in the article/supplementary material, further inquiries can be directed to the corresponding authors.
